# Pay for performance – motivation to succeed in Advanced Trauma Life Support courses – a question of background or funding?

**DOI:** 10.3205/iprs000118

**Published:** 2017-12-05

**Authors:** Roman Klein, Wolfgang Armbruster, Martin Grotz, Bernd Höner, Matthias Münzberg, Paul Alfred Grützner, Christoph Georg Wölfl

**Affiliations:** 1 Clinic of orthopedic surgery, trauma surgery and sports traumatology, Krankenhaus Hetzelstift, Neustadt an der Weinstrasse, Germany; 2 Clinic of orthopedic surgery and trauma surgery, Kreiskrankenhaus Bergstraße, Heppenheim, Germany; 3 MDKN – Geschäftsbereich Behandlungsfehler, Hannover, Germany; 4 SRH-Hochschule Heidelberg, Department for social studies and law, Heidelberg, Germany; 5 BG Trauma Center Ludwigshafen, Department of trauma surgery and orthopedics, Ludwigshafen, Germany

**Keywords:** Advanced Trauma Life Support, ATLS, motivation, incentives, test performance

## Abstract

**Objective:** To correlate students’ performance with their professional background and motivation to take part in Advanced Trauma Life Support (ATLS) courses. We base our analysis on the self-determination theory that differentiates intrinsic (ambition to perform by individual itself) from extrinsic motivation (incentive by external stimuli).

**Design:** We present a non-blinded, monocentric, non-randomized descriptive study of 376 students taking part in an ATLS course at one course site in Germany. Part of a two-day ATLS course are two written tests; we correlate test scores with background information provided by the students in a questionnaire of 13 items (age, sex, adress, board certification, specialty, subspecialty, position, hospital level of care, hospital operator and hospital participation in trauma network, motivation, funding source, condition of funding).

**Setting:** The students were recuited at the BG Trauma Center Ludwigshafen (Germany), a large 528-bed trauma center and one of 13 ATLS course sites in Germany.

**Participants:** 449 ATLS course students taking part in ATLS courses at the above-mentioned course site from February 2009 to May 2010 were sent a questionnaire asking for their background. All 449 course students were eligible to participate. 376 (83.7%) questionnaires were returned, pre- and post-test results of all students aquired and included into our calculations. 312 (83%) were male and 64 (17%) female. The majority (59.3%) of recruited students came from trauma surgery, 21.8% from anesthesiology, 8% from general surgery, 4% from abdominal surgery, 0.5% from vascular or thoracic surgery each and 5.9% from other specialties.

**Results:** Neither age, sex, subspecialty, hospital level of care, hospital operator, or hospital participation in trauma network played a role with respect to motivation or test results. The high degree of intrinsic motivation of consultants (92.3%) had no impact on their test results. Anesthesiologists were higher motivated (75.6% intrinsically motivated) in contrast to all surgical colleagues (63.6%), which showed significant differences in the pre- (89.8% vs. 85.3%, p=0.03) but not the post-test. Of all 13.6% students who were self-payers, 94.1% were intrinsically motivated; the 86.2% whose course fee was accounted for were less likely to be intrinsically motivated (63.9%). Sponsoring however did not have a negative impact on test results. Conditional funding (sponsored only on passing both tests) was detrimental to motivation: 0% of these individuals were intrinsically motivated and they scored significantly lower (82.5%) than all other students in the post-test (86.9%, p=0.002). Overall, intrinsically motivated students overtopped extrinsically motivated students in the post-test (88.0% vs. 83.4%, p<0.001).

**Conclusions:** ATLS course participation is not compulsory for medical doctors in Germany. Intrinsic motivation to take part in these courses is a key prerequisite to increase performance, irrespective of the background of the students. Intrinsically motivated students are ready to invest into their education and vice versa. Conditional funding (course fee only sponsored on passing the course) evokes no intrinsic motivation at all and causes worse results.

## Zusammenfassung

**Ziele:** Korrelation der Testergebnisse von Teilnehmern an Advanced Trauma Life Support (ATLS)-Kursen mit deren fachlichen Hintergrund und Motivation. Die Auswertung basiert auf der Selbstbestimmungstheorie, die zwischen intrinsischer (Eigenantrieb) und extrinsischer Motivation (externe Anreize) unterscheidet.

**Material und Methoden:** Wir stellen eine unverblindete, monozentrische, nicht-randomisierte, deskriptive Studie mit 376 Teilnehmern vor. Die Teilnehmer wurden an der Berufsgenossenschaftlichen Unfallklinik in Ludwigshafen, einem großen Trauma-Zentrum mit 528 Betten und einem von 13 ATLS-Kursorten in Deutschland, rekrutiert.

Teil eines jeden Zweitages-ATLS-Kurses sind zwei schriftliche Tests (Eingangs- und Abschlusstest). Wir korrelierten die hier erzielten Testergebnisse mit Hintergrundinformationen eines Fragebogens mit 13 Items (Alter, Geschlecht, Adresse, Facharztstatus, Facharztdisziplin, Zusatzbezeichnung, Position, Versorgungsstufe des Krankenhauses, Krankenhausträger, Partizipationsstatus im Trauma-Netzwerk, Motivation, Geldquelle, Erfolgsabhängkeit der Fremdfinanzierung).

Insgesamt 449 Kursteilnehmer zwischen Februar 2009 und Mai 2010 erhielten den o.g. Fragebogen mit der Einladung zum Kurs per Post. Alle 449 Kursteilnehmer waren in unserer Studie teilnahmeberechtigt. 376 (83,7%) der Fragebögen wurden retourniert, die Ergebnisse von Eingangs- und Abschlusstest aller Studienteilnehmer erfasst und in unsere Berechnungen einbezogen. 312 (83%) waren Männer und 64 (17%) Frauen. Die Mehrzahl (59,3%) der rekrutierten Studienteilnehmer waren Unfallchirurgen, 21,8% Anästhesisten, 8% Allgemeinchirurgen, 4% Viszeralchirurgen, 0,5% jeweils Gefäß- und Thoraxchirurgen und 5,9% Ärzte aus anderen Disziplinen.

**Ergebnisse:** Weder Alter, Geschlecht, Zusatzbezeichnung(en), Versorgungsstufe, Krankenhausträger noch Partizipationsstatus in Bezug auf das Trauma Netzwerk spielten eine Rolle in Bezug auf Motivation oder Testergebnisse. Den größten Anteil intrinsisch motivierter Teilnehmer verzeichneten Chefärzte (92,3%), jedoch ohne Implikationen auf deren Testergebnisse. Der Anteil intrinsisch motivierter Anästhesisten war höher (75,6%) im Vergleich zu allen Chirurgen (63,6%); dies schlug sich in signifikant besseren Ergebnissen im Eingangs- (89,8% vs. 85,3%, p=0,03), jedoch nicht im Abschlusstest nieder.

Von den 13,6% aller Selbstzahler waren 94,1% intrinsisch motiviert. Nur 63,9% der 86,2% Fremdfinanzierten waren intrinsisch motiviert. Der Aspekt der Finanzierung der Kursgebühr hatte keine Auswirkungen auf die Testergebnisse. Die *erfolgsabhängige* Finanzierung der Kursgebühr hatte dabei jedoch einen deutlich negativen Effekt in Bezug auf die Motivation der betroffenen Teilnehmer: niemand aus dieser Gruppe war intrinsisch motiviert und sie erzielte schlechtere Ergebnisse (82,5%) im Abschlusstest im Vergleich zu allen anderen Teilnehmern (86,9%, p=0,002). Generell erreichten alle intrinsisch motivierten Teilnehmer im Abschlusstest bessere Ergebnisse als extrinsisch motivierte (88,0% vs. 83,4%, p<0,001).

**Schlussfolgerungen:** Die Teilnahme an ATLS-Kursen ist bislang für Ärzte in Deutschland nicht verpflichtend. Intrinsische Motivation zur Teilnahme an diesen Kursen ist ein Schlüsselfaktor in Bezug auf die Testergebnisse, unabhängig vom fachlichen Hintergrund. Intrinsisch motivierte Teilnehmer sind zu Investitionen in Ihre Ausbildung bereit und vice versa. Erfolgsabhängige Finanzierung der Kursgebühr macht jegliche intrinsische Motivation zunichte und hat schlechtere Testergebnisse zur Folge.

## Definitions

**Hospitals in Germany – levels of care**

- **General hospitals – level III trauma centers**
  Hospitals that provide regional care in internal medicine and surgery. Departments of other medical specialties are optional.
- **Hospitals for specialised medical services – level II trauma centers**
  Hospitals that dispose of additional specialties like gynecology and obstetrics, ophthalmology, ear nose throat medicine, orthopedics, pediatrics and urology that serve patients from a larger community and area.
- **Maximum care hospitals – level I trauma centers**
  Hospitals (e.g. university hospitals) that offer care beyond of the above-mentioned specialised services providing highly-differentiated medical/surgical specialities.

## Introduction

### Advanced Trauma Life Support (ATLS) in Germany

Dealing with severly injuried patients is an old field in medicine. *Structured* training of doctors to treat these conditions however is new in medical history. The inciting event for implementation arose from the dire insights of Jim Styner, M.D. The surgeon crashed his plane in 1976, leaving his wife dead on the spot and his children injured. The poor intrahospital treatment of his family and himself caused Styner to devise and implement training to improve acute trauma care (advanced trauma life support, ATLS) [1]. The concept of ATLS is to “treat first what kills first”: the patient is examined and *immediately* treated by a scheme that follows an alphabetical order: A: airway and cervical spine immobilisation, B: breathing, C: circulation, D: disability, and E: exposure and environmental control. This ensures that vital diagnostic and therapeutic measures are provided in a timely fashion.

In the United States, an ATLS course program was first launched in 1978, but it was not until 2003 that ATLS training was introduced in Germany [2]. To date, over one million doctors participated in ATLS courses worldwide [3]. Moreover, so called trauma networks were founded in 2006 by the German Society for Trauma Surgery to optimize and standardize care for the injured [4], [5]. Among other prerequisites, participating hospitals are required to predispose of ATLS-providing medical doctors (i.e. ATLS course graduates) [6], [7]. Although it has been shown that structured training improves patient care by speeding up vital diagnostic and therapeutic measures [8], [9], ATLS course outcomes correlate well with established courses like the OSCE [10] and German courses are excellently evaluated by students [11], participation is not compulsory for all medical doctors in Germany until today. 

### Course structure

ATLS courses are available at 13 centers in Germany. The course takes two days on weekends and costs approximately 1400 €. A maximum of 16 students are taught by lectures and practice lessons in small groups (usually of four). Before the course begins, students are provided with a textbook [12] and a 40-item multiple-choice “*open book*” (“pre”) test to be handed in on arrival at the course site. This test consists of 40 items of 5 answers, one correct answer each. 32 questions or more (≥80%) are to be answered correctly. After the course, there is a *supervised* post-tests with the same structure and pass/fail-criteria. Moreover, an additional practice test is to be passed (pass/fail only); the results of the latter test are not taken into account here.

### Motivation

Motivation is a colloquial term that has undergone scientific research from the 1970s onwards. It is defined as the process that makes a person increase efforts to achieve a defined goal [13].

Several approaches have been pursued to better define motivation. The self-determination theory [14] – on which we base this work here – differentiates motivation depending on the degree of self-determination. As personal interest and goals are the basis of motivation [15], it has been subdivided into: 

*Intrinsic* motivation is based merely on the interst of the individual itself without extrapersonal factors. It has the highest degree of self-determination. *Extrinsic* motivation on the other hand is not (or to a certain extent) based on an individual’s own motives.

Reeves, Deci and Ryan [15] further differentiate (listed by increasing degree of self-determination):

1. amotivation (lacking the intention to act)
2. extrinsic motivation:
  a. external regulation (gain reward/avoid punishment)
  b. introjected regulation (conduct is regarded as right or important by community/society)
  c. identified regulation (conduct is regarded as right and important by person himself)
  d. integrated regulation (identifies himself with norms and goals)
3. intrinsic regulation (motivation emerges spontaneously).

In this work here, we only differentiate between extrinsic motivation (questionnaire: “sent by superior/institution”) or intrinsic motivation (questionnaire: “own interest”).

### Objective of this study

After a considerable time of organizing ATLS courses, ATLS course directors noticed a decline in test results. In light of the above-mentioned criteria for hospitals to participate in the trauma network and doctors to be trained with or without compensation of the course fee, we became interested in the correlation of motives and motivational aspects with test performance. 

## Material and methods

To test the impact of motivation on the outcome in the ATLS course, we devised a questionnaire that was sent to future students before the course started and handed in on arrival at the course site. Taking part in our study was entirely voluntary.

### Participants

**Figure 1:**
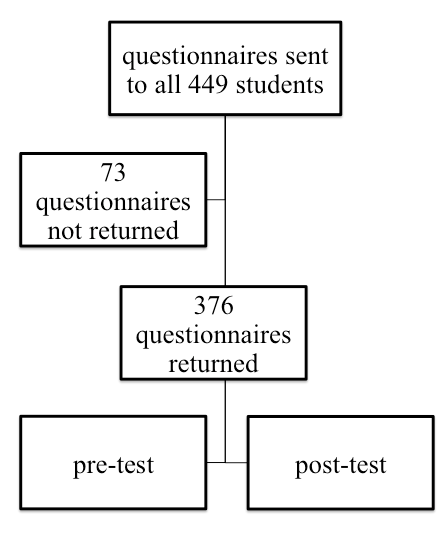
Study design Questionnaire return was required for inclusion in our study. Return/recruitment rate of students was 83.7%. The test results of pre- and post-test were correlated with the background-data provided in the questionnaire.

From February 2009 to May 2010 (28 courses) of 449 ATLS students in total, 376 (83.7% recruited, 312 male / 64 female) were recruited for this study at the BG Trauma Center Ludwigshafen, Germany (see Figure 1 (Fig. 1)). The composition of the students’ background is represented in Table 1 (Tab. 1). 

### Questionnaire

**Figure 2:**
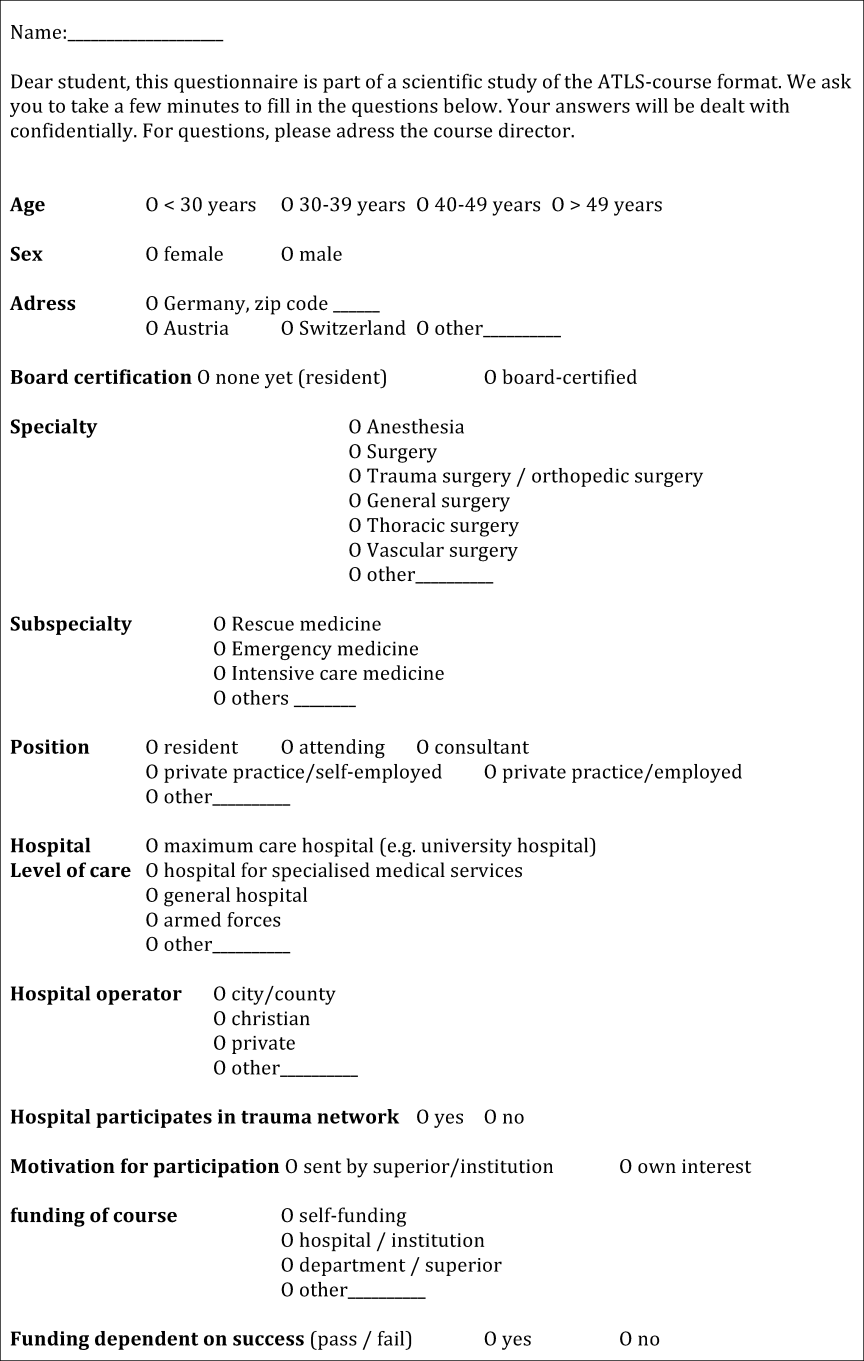
Questionnaire sent to students (originally in German, translated into English) 13 items had to be answered to provide background- and motivational aspects to be correlated with test performance of every student.

The questionnaire was subdivided into 13 items (Figure 2 (Fig. 2)). After indicating their sex (female, male), students were to fill in the age group (<30 years, 30 to 39 years, 40 to 49 years and >49 years) they belonged to. Furthermore, the place of residence (Germany including zip-code, Austria, Switzerland or other) was of interest. To be able to evaluate level of training, board certification (yes or no), specialty (anesthesiology, surgery, trauma surgery/orthopedic surgery, general surgery, thoracic surgery, vascular surgery and other), subspecialty (rescue medicine, emergency medicine, intensive care and other) as well as position (resident, attending, consultant, private practice/self-employed and private practice/employed and other) were to be indicated. It was furthermore evaluated of which level of care the home institution was (maximum care hospital, hospital for specialised medical services, general hospital, armed forces hospital and other; see definitions above); its operator (city/county, christian, private and other) and participation status with respect to the trauma network (yes or no) had to be specified. Of utmost interest was if the students were either sent by their superior(s) or participated due to his/her own interest. They had to indicate the source of funding (self-payers, hospital/institution, department/superior and other) and if the funding was granted only depending on passing the course requirements. The name and zip code were used to identify and correlate questionnaire content with pre- and post-test score of every student.

### Pre- and post-tests

After handing in the questionnaire and pre-test, the course started as planned. The post-test concluded the two-day course. All test results of pre- and post-tests of all 376 students were obtained, included in our observation and correlated with answers provided in the questionnaire.

### Statistical analyses

The data set was subjected to the Kolmogorov-Smirnov test and proved not to obey to Gaussian distribution. Therefore, non-parametric tests had to be employed to calculate statistical significance for two variables: Mann-Whitney U test, Wilcoxon U test, Kolmogorov-Smirnov Z-test, Wald-Wolfowitz sequence test and Moses test. Whenever more than two variables were considered, Kruskal-Wallis test or median test were used. As the question for intrinsic motivation in the questionnaire was a binary item, the data set yielded a scale of nominal level. A comparison of subgroups with respect to motivation (e.g. attendings vs. consultants) can therefore only be descriptive.

Whenever applicable, statistical significance is indicated by *: p<0.05, **: p<0.01, ***: p<0.001.

### Ethics

The ethics commission of the Landesärztekammer Rheinland-Pfalz (State Medical Council Rhineland-Palatinate, Germany) approved of the study protocol. The processing number allocated is 837.032.11 (7574). 

## Results

### All pre – post

To establish an effect of the course success, we analysed the test results of all 376 students before (pre-test) and after (post-test) the course. They attained 86.4% in the pre- and 86.5% in the post-test, which did not show statistical significance. 

### Age groups

9.6% were younger than 30 years, 46.5% 30 to 39 years, 32.4% 40 to 49 years and 11.4% older than 40 years old. Students younger than 30 years (69.4%) and 30 to 39 years of age (70.3%) were more likely to be intrinsically motivated compared to both 40 to 49 (65.1%) and more than 49 years of age (64.8%). There was however no impact of motivation on test results (data not shown).

### Sex

The majority (83%) were male and only 17% female. Although men were more likely to be intrinsically motivated (68.6%) than women (64.1%), female students scored better in pre- (85.9% vs. 88.9%) and post-tests (86.4% vs. 87.5%). The results did not prove to be statistically significant.

### Board certification

Completion of board certification (59% were board-certified physicians) was of little implication with respect to intrinsic motivation (no: 85.0% vs. yes: 83.0%) and no implication with respect to test results: pre (86.7% vs. 86.2%, not significant) and post (87.3% vs. 86.0%, not significant).

### Specialty and subspecialty

**Figure 3:**
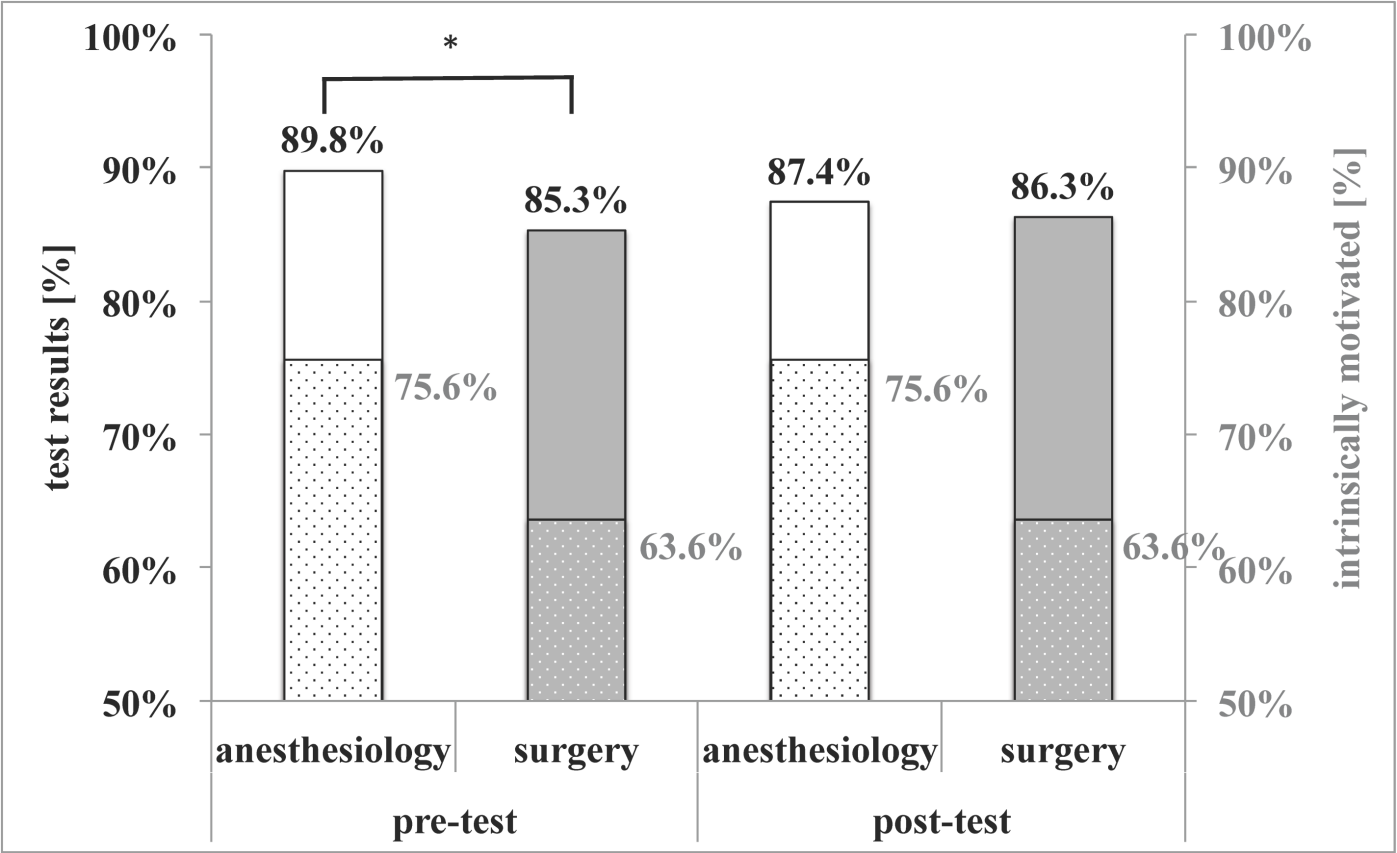
Test results of anesthesiologists vs. all surgeons Of all seven medical specialties considered, anesthesiologists achieved the best results in the pre-test, but not post-test. Black numbering, uniformely colored bars and left y-axis: test results [%]. Grey numbering, dotted bars and right y-axis: % of intrinsically motivated students. Statistical significance indicated by *: p<0.05, **: p<0.01, ***: p<0.001 in the Kolmogorov-Smirnov test.

The majority (59.3%) of students’ specialty was trauma surgery, 21.8% anesthesiology, 8% general surgery, 4% abdominal surgery, 0.5% vascular or thoracic surgery each and 5.9% indicated other specialties. Anesthesiologists were highly intrinsically motivated (75.6%) in contrast to all surgeons taken together (63.6%). This proved to be influencial with respect to test results (Figure 3 (Fig. 3)): they scored significantly better than all surgical specialties taken together (89.8% vs. 85.3%, p=0.03, Kolmogorov-Smirnov test) in the pre-test. This head-start was however lost in the post-test (87.4% vs. 86.3%, not significant). Subspecialty had no influence on test outcomes.

### Position

56.6% were residents, 33.2% attendings, 6.9% consultants working in the hospital. 0.3% worked in a private practice, employed or self-employed each. 2.4% did not indicate their position.

Consultants were the most likely to be intrinsically motivated (92.3%) in contrast to attendings (64.0%) and residents (66.2%). The test scores of the mentioned groups did however not differ statistically in either pre- or post-tests (data not shown). 

### Hospital

Neither the size/level of care, participation in the trauma-network (yes 88.8%, no 11.2%) or hospital operator played a role in motivational aspects or test results (data not shown).

### Funding of course fee

The 13.6% of all students who indicated to be self-payers were better motivated (94.1%) in contrast to the 86.2% whose course fee was paid for (63.9%). The former attained 86.9% in the pre-test and 88.2% in the post-test, but did not score higher than those who could rely on other sources of funding.

**Figure 4:**
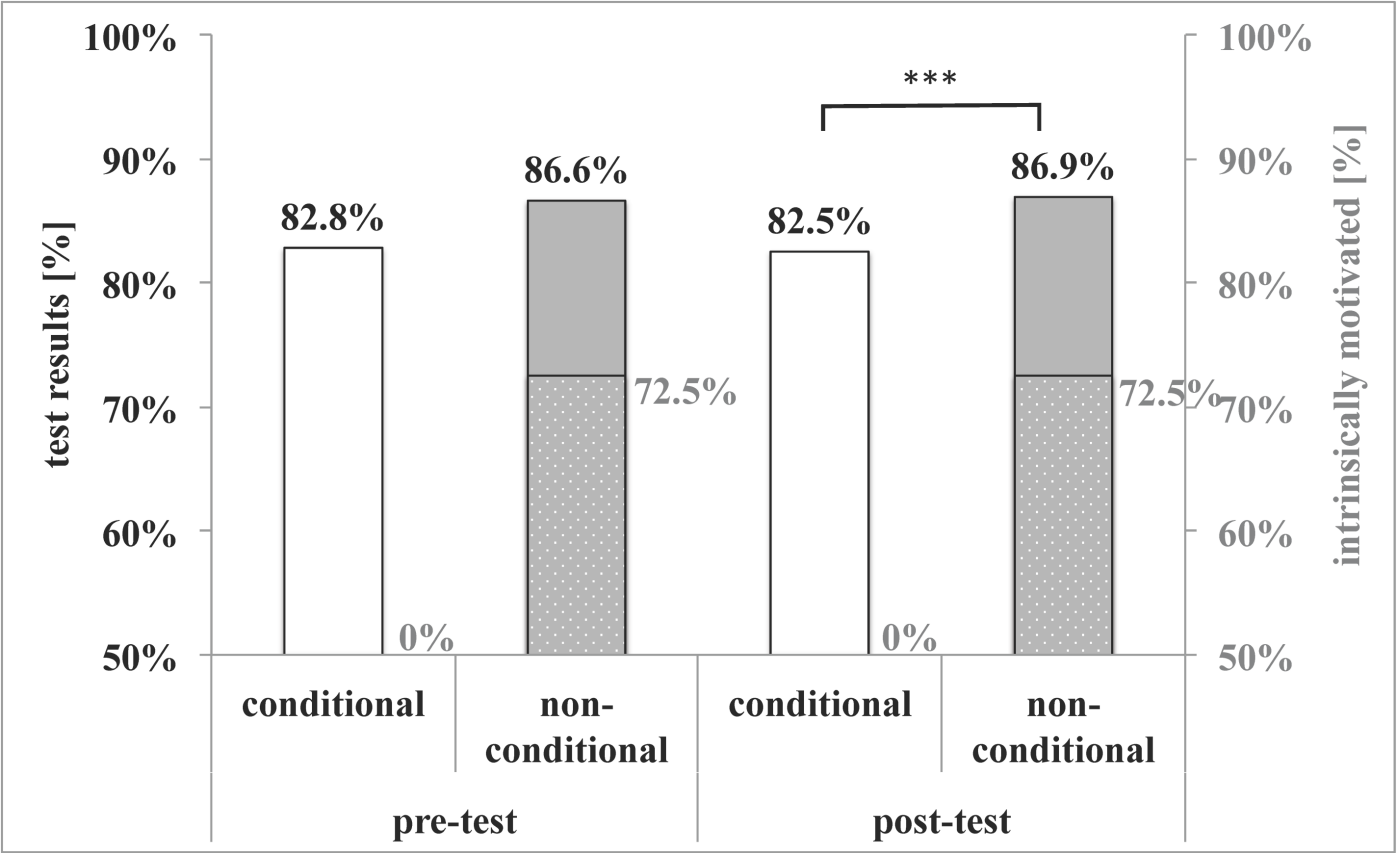
Test results of students conditionally and non-conditionally funded Conditional sponsoring of the course fee was detrimental to motivation and test results of the post-test. Black numbering, uniformely colored bars and left y-axis: test results [%]. Grey numbering, dotted bars and right y-axis: % of intrinsically motivated students. Statistical significance indicated by *: p<0.05, **: p<0.01, ***: p<0.001 in the Kolmogorov-Smirnov test.

Conditional funding (course sponsored only on passing both tests) was detrimental (Figure 4 (Fig. 4)) to motivation (0% intrinsically motivated of the 7.2% sponsored conditionally vs. 72.5% intrinsically motivated of the 86.2% non-conditionally funded) and test results: pre (conditional 82.8% vs. non-conditional 86.6%; not significant) and post (conditional 82.5% vs. non-conditional 86.9%; p=0.002, Kolmogorov-Smirnov test).

### Motivation 

**Figure 5:**
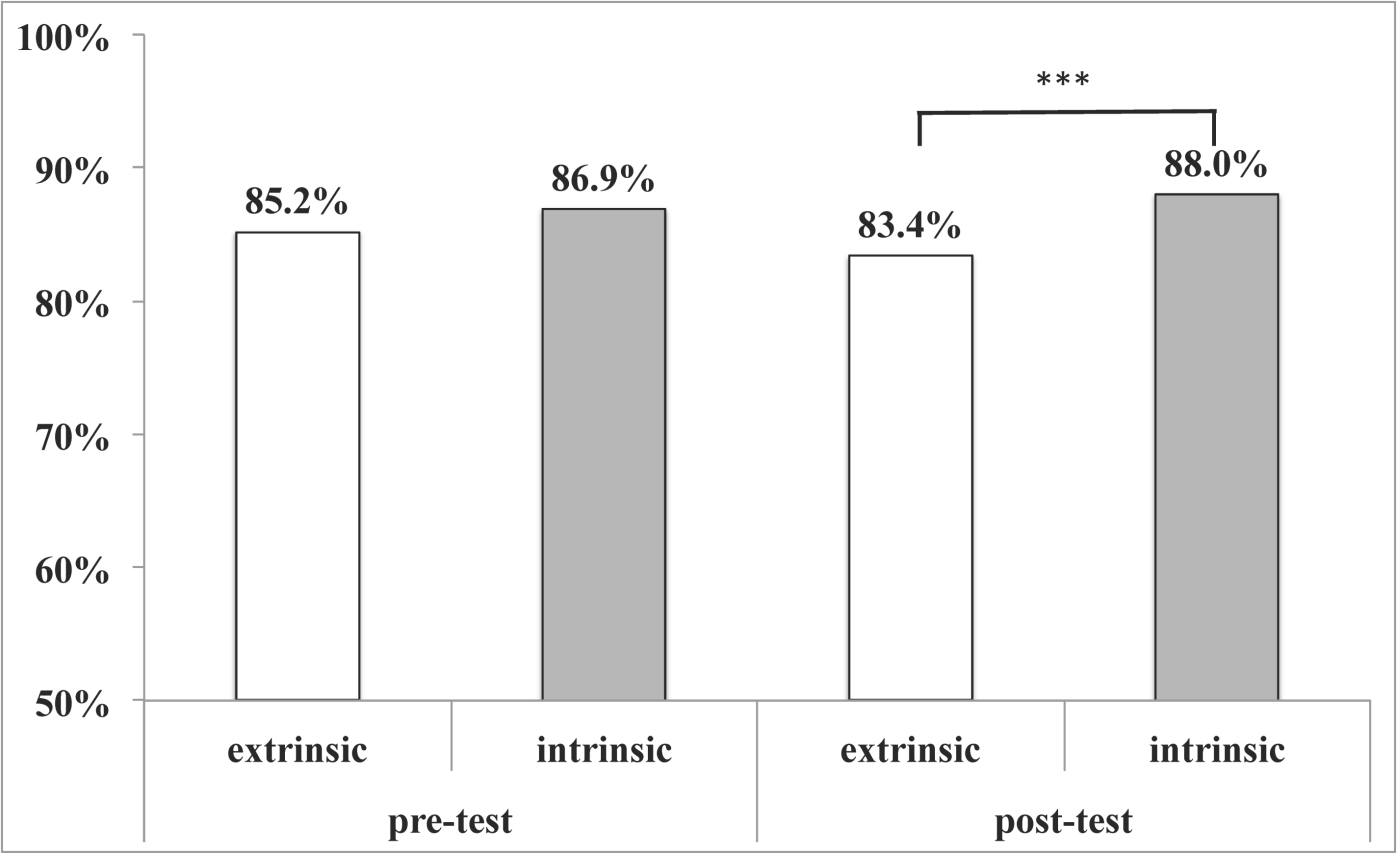
Impact of intrinsic motivation on test results The majority of students was intrinsically motivated, which helped with test results. Statistical significance indicated as result of the Kolmogorov-Smirnov test. Statistical significance indicated by *: p<0.05, **: p<0.01, ***: p<0.001 in the Kolmogorov-Smirnov test.

Intrinsically motivated students made up the majority (67.8%) of all students. Their test results were better in pre- (86.9% vs. 85.2%, not significant) and post-tests (88.0% vs. 83.4%, p<0.001, Kolmogorov-Smirnov test, see Figure 5 (Fig. 5)).

## Conclusions

Structured training of doctors with role play and lectures to deal with severely injured patients has been proven to be beneficial with respect to morbidity and mortality. It helps to provide an organized approach in the trauma room and is perceived as helpful by physicians [16], [17], [18]. The ATLS program was introduced to improve trauma care in Germany in 2003. The implementation of trauma networks in Germany in 2006 now requires participating hostpitals to dispose of ATLS-trained physicians to become a certified trauma center [7]. This is reflected by the high percentage (88.8%) of students whose home institution was a trauma center in our study. Whereas in other countries like England, ATLS courses have become a compulsory part of a physician’s training [19], medical doctors in Germany are not required to be ATLS providers unless they work in a trauma center.

In the present study, we analysed the background of ATLS course students and correlated motivation and test results of a cohort of 376 students from February 2009 to May 2010 at one of the 13 course sites in Germany. With respect to the obligation to fulfill the trauma center requirements and the funding of the course fee (an equivalent of approximately half a resident’s monthly net salary), we correlated motivational and professional background with test results. 

It was conspicuous that more than half of all students of our study were under 40 years of age. One might conclude that doctors in the beginning of their career have a higher incentive to perform as motivation is known to be age-dependant [20]. Although we observed a higher percentage of intrinsically motivated younger students, neither age, sex, nor position or board-certification status played a role with respect to test results. Likewise, trauma center status or level of care of the respective hospital had no implication on motivation or performance of students.

It has been shown before that intrinsic motivation improves quality of preparation [21]. Looking at the different clinical fields that students were coming from, anesthesiologists were the most likely to be intrinsically motivated of all specialties and overtopped surgical colleagues’ results in the pre-, but not the post-test. As the pre-test is an open book test at home before the course, its results represent mostly diligence and preparation. Therefore, the surveilled post-test should be regarded as a true test of knowledge as well as a measure to assess the training effect of the ATLS course. The fact that pre- and post-test results did not differ statistically in all students is due to the very same fact: the same results could be achieved without textbook reference and should not be interpreted as lack of course outcome with respect to knowledge taught.

Overall, approximately two thirds (67.8%) of students were intrinsically motivated which showed in the performance in the post-test where they overtopped the “forced” (extrinsically motivated) ones. Intrinsic motivation has been shown to increase performance the best [13], but our questionnaire also took into account incentives to perform as provided by superiors: The course fee of 7% of students was only accounted for only on passing the ATLS course. None of these 27 doctors felt intrinsic motivation to pass and scored significantly lower than those wo received unconditional sponsoring of the course fee. As no surprise, this form of extrinsic modulation of conduct comes with the lowest level of self-determination. A meta-analysis of 92 studies on motivational aspects stated that modification of conduct by monetary rewards is short-lived [22]. 

In conclusion, we have shown that motivation is of utmost importance for the performance in ATLS courses and probably medical training in general. If intrinsically motivated, students invest a considerable amount of money and perform well. Neither sponsoring – or worse – sponsoring only in case of success can make up for an individual’s own will to perform. 

### Highlights

- Intrinsic motivation is a key prerequisite to increase performance, irrespective of the background of the students.
- Intrinsically motivated students are ready to invest into their education and vice versa.
- Conditional funding (course fee only reimbursed on passing the course) evokes no intrinsic motivation at all and causes bad results.

## Competing interests

Martin Grotz, Matthias Münzberg and Christoph Wölfl were ATLS course directors when this study was performed. Financial conflicts of interest: none. 

## Figures and Tables

**Table 1:**
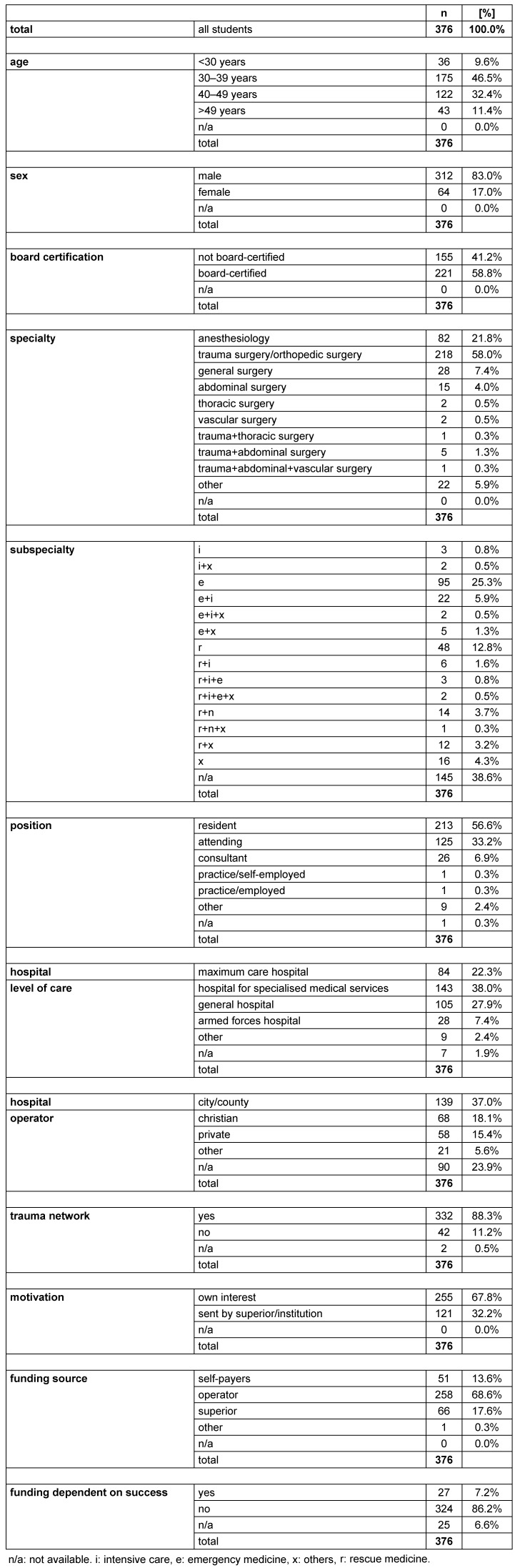
Composition of students of our study
